# Identification of Chinese medicine syndromes in persistent insomnia associated with major depressive disorder: a latent tree analysis

**DOI:** 10.1186/s13020-016-0076-y

**Published:** 2016-02-12

**Authors:** Wing-Fai Yeung, Ka-Fai Chung, Nevin Lian-Wen Zhang, Shi Ping Zhang, Kam-Ping Yung, Pei-Xian Chen, Yan-Yee Ho

**Affiliations:** School of Chinese Medicine, University of Hong Kong, Pokfulam Road, Hong Kong SAR, China; Department of Psychiatry, University of Hong Kong, Hong Kong SAR, China; Department of Computer Science and Engineering, The Hong Kong University of Science and Technology, Hong Kong SAR, China; School of Chinese Medicine, Hong Kong Baptist University, Hong Kong SAR, China; Department of Psychology, The Chinese University of Hong Kong, Hong Kong SAR, China; Department of Psychology, The University of Hong Kong, Hong Kong SAR, China

**Keywords:** Chinese medicine syndrome, Zheng, Insomnia, Latent tree analysis, Latent tree model, Patterns

## Abstract

**Background:**

Chinese medicine (CM) syndrome (*zheng*) differentiation is based on the co-occurrence of CM manifestation profiles, such as signs and symptoms, and pulse and tongue features. Insomnia is a symptom that frequently occurs in major depressive disorder despite adequate antidepressant treatment. This study aims to identify co-occurrence patterns in participants with persistent insomnia and major depressive disorder from clinical feature data using latent tree analysis, and to compare the latent variables with relevant CM syndromes.

**Methods:**

One hundred and forty-two participants with persistent insomnia and a history of major depressive disorder completed a standardized checklist (the Chinese Medicine Insomnia Symptom Checklist) specially developed for CM syndrome classification of insomnia. The checklist covers symptoms and signs, including tongue and pulse features. The clinical features assessed by the checklist were analyzed using Lantern software. CM practitioners with relevant experience compared the clinical feature variables under each latent variable with reference to relevant CM syndromes, based on a previous review of CM syndromes.

**Results:**

The symptom data were analyzed to build the latent tree model and the model with the highest Bayes information criterion score was regarded as the best model. This model contained 18 latent variables, each of which divided participants into two clusters. Six clusters represented more than 50 % of the sample. The clinical feature co-occurrence patterns of these six clusters were interpreted as the CM syndromes *Liver qi stagnation transforming into fire, Liver fire flaming upward, Stomach disharmony, Hyperactivity of fire due to yin deficiency, Heart*–*kidney noninteraction, and Qi deficiency of the heart and gallbladder.* The clinical feature variables that contributed significant cumulative information coverage (at least 95 %) were identified.

**Conclusion:**

Latent tree model analysis on a sample of depressed participants with insomnia revealed 13 clinical feature co-occurrence patterns, four mutual-exclusion patterns, and one pattern with a single clinical feature variable.

**Electronic supplementary material:**

The online version of this article (doi:10.1186/s13020-016-0076-y) contains supplementary material, which is available to authorized users.

## Background

Chinese medicine (CM) syndrome (*zheng*) differentiation is a diagnostic summary of the pathological changes of a disease state based on an individual’s symptoms and signs, including pulse form and tongue appearance [1]. CM patterns of bodily disharmony can be described in terms of eight basic parameters: *yin* and *yang*, *external* (*biao*) and *internal* (*li*), *hot* (*re*) and *cold* (*han*), and *excess* (*shi*) and *deficiency* (*xu*). Additional systems differentiate syndromes in terms of *qi*, *blood* (*xue*), *body fluids* (*jinye*), and *organs* (*zang fu*) [2].

For any given disease, there may be different presentations of symptoms and signs; therefore, different CM treatments may be given for the same disease. Conversely, the same CM treatment may be used for different diseases if they share the same pattern. Individualized treatment can maximize the effectiveness of CM [3]. Today, CM syndrome definitions and criteria are based on expert opinions; however these are imprecise, may vary across CM textbooks [4–6], and have sometimes been poorly described in previous clinical studies [7]. Additionally, it is not clear whether CM syndromes correspond to real-world entities or are merely subjective notions [8].

Attempts have been made to correlate CM syndromes with biomedical indexes. One study found that bone mineral density was lower in participants with chronic obstructive pulmonary disease and *kidney deficiency* (*shenxu*) than in those without *kidney deficiency* [9]. A study examining gene expression profiles and CM syndromes found that rheumatoid arthritis and *cold* syndrome symptoms were related to the toll-like receptor signaling pathway, whereas *heat* syndrome symptoms were associated with the calcium signaling pathway, cell adhesion molecules, the peroxisome proliferator-activated receptor signaling pathway, and fatty acid metabolism [10]. Despite these encouraging findings, it remains unclear whether current CM syndrome classification truly reflects various pathological states.

As CM syndrome classification is based on co-occurrence of manifestation profiles, Zhang et al. [11, 12] used a latent tree analysis (LTA) to examine co-occurrence patterns. Latent factors are identified by the presence or absence of specified clinical features of a particular disease or condition. The LTA is based on latent tree models (LTMs). An LTM describes the relationships between observed variables (clinical feature variables) and unobserved variables (latent variables) [13]. It has a tree structure in which the observed variables are located at the leaf nodes and the latent variables are located at the internal nodes. Using the LTA, latent variables are introduced to explain co-occurrence patterns or mutual-exclusion patterns, and these are built up to form a tree. Zhang et al. [11] used LTA with a group of participants with *kidney deficiency.* The latent variable, consisting of intolerance of coldness, cold lumbus and back, and cold limbs, could be interpreted as *kidney yang* failing to warm the body.

LTA has been used in a number of other studies. Xu et al. [8] used LTA to analyze questionnaire data from participants with cardiovascular diseases and Zhao et al. [14] performed LTA on data from a symptom checklist of manifestation patterns in participants with depression. In both studies, LTA yielded a number of latent variables that corresponded to CM syndromes. Chen et al. [15] attempted to construct LTA models of *kidney yang deficiency (shenyang xu)* and *kidney yin deficiency* with an existing data set from healthy middle-aged women, but the models have yet to be examined in a participant sample. Although the LTA method has yet to be used clinically, it is the first step in summarizing co-occurrence patterns, which can be used to construct an applicable algorithm.

This study aims to identify co-occurrence patterns in participants with persistent insomnia and major depressive disorder from clinical feature data using LTA, and to compare the latent variables with relevant CM syndromes. The participants’ symptoms and signs, including tongue and pulse features, were assessed by a standardized symptom checklist: the Chinese Medicine Insomnia Symptom Checklist, specially developed for CM syndrome diagnosis for insomnia.

## Methods

Participants were recruited from four regional psychiatric outpatient clinics in Kowloon Hospital, Kwai Chung Hospital, Queen Mary Hospital, and United Christian Hospital, all in Hong Kong. The participants were subjects of a randomized controlled trial of acupuncture for residual insomnia with major depressive disorder [16]. The recruitment period was from May 2011 to August 2013 and 975 participants were initially screened for eligibility. Written informed consent was given by all participants [16]. The inclusion criteria were (1) ethnic Chinese; (2) aged 18 years or above; (3) previous diagnosis of major depressive disorder based on the criteria from the *Diagnostic and Statistical Manual of Mental Disorders*, fourth edition, text revision edition (DSM-IV-TR), as assessed by a clinician using the Structured Clinical Interview for DSM-IV-TR [17]; (4) full or partial remission of major depressive disorder based on a 17-item Hamilton Depression Rating Scale [18] score ≤18 at screening and baseline; (5) complaint of insomnia for at least 3 months; and (6) an Insomnia Severity Index [19, 20] score ≥15 at screening and baseline.

### Procedures

All procedures used in the present study were reviewed and approved by the local institutional review board. The research ethic approval (Additional file 1), research protocol (Additional file 2), and patient informed consent form (Additional file 3) are listed as additional files in this article. We used the Chinese Medicine Insomnia Symptom Checklist (Additional file 4) which was developed based on our previous systematic review [21], to systematically collect clinical data from the participants. In our previous review, we summarized the manifestation profiles reported in 103 studies on CM syndromes in participants with insomnia and identified 52 sleep-related symptoms, 169 non-sleep-related symptoms, 19 tongue features, and seven pulse signs that were described in the 10 most common CM syndromes. After grouping similar terms, a symptom checklist with 93 items, including 13 sleep-related symptoms, 62 non-sleep-related symptoms, 11 tongue features, and seven pulse items, was developed. In the present study, CM practitioners with at least 3 years’ clinical experience went through the checklist with the participants to assess the presence or absence of the clinical features. One hundred and forty-two participants completed the Chinese Medicine Insomnia Symptom Checklist.

### Data analysis

The symptom data were analyzed by the Extension Adjustment Simplification until Termination (EAST) algorithm [22] for LTA, available from Lantern software [23]. The EAST algorithm is a search-based algorithm aimed at finding the LTM with the highest Bayes information criterion (BIC) score [24]. It starts with the simplest LTM with one dichotomous latent variable. At each search step, it considers various ways of modification, by introducing a new latent variable, increasing the number of states for a latent variable, or adjusting connections among existing variables. The different models are evaluated by the BIC score. As each model’s BIC score is computed, its probabilistic parameters are estimated by the Expectation–Maximization (EM) algorithm [25]. As the search progresses, the models become more complex and the BIC scores increase gradually until the BIC score ceases to improve and the best model obtained at the last step is taken as the final model for the data set.

To determine the CM syndrome connotations of the latent variables, one author (WFY), who is a registered CM practitioner, consulted with another author (SPZ) to compare the clinical feature variables identified under each latent variable with the clinical features described in the 10 most common CM syndromes associated with insomnia, as summarized in a previous systematic review [21]. However, as this was the first-step of LTA to explore the co-occurrence pattern in a clinical sample with insomnia, we did not validate or compare the results with other non-LTA models.

## Results

**Fig. 1:**
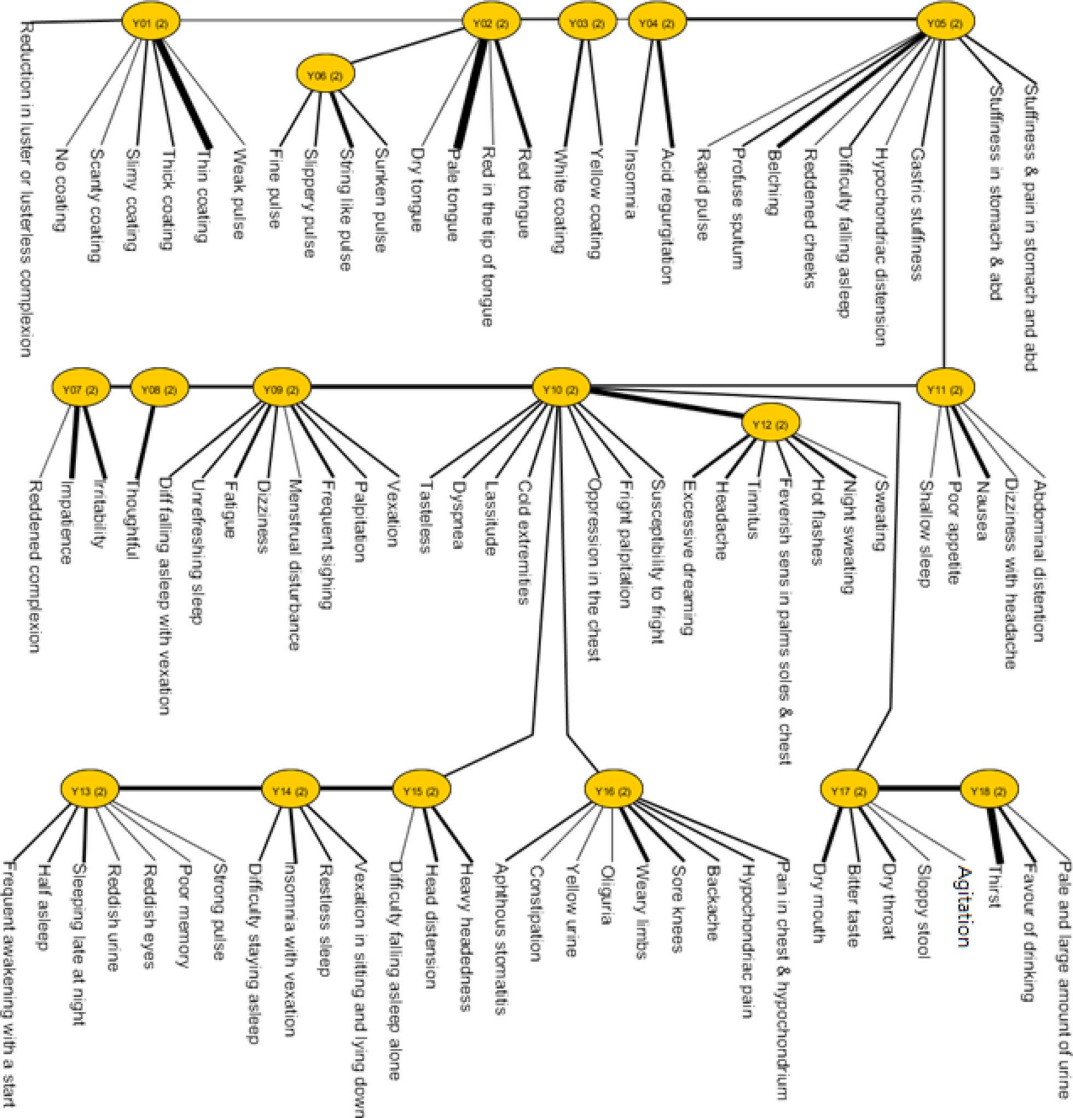
The structure of the model obtained by latent tree analysis on the depressed patients with persistent insomnia data set

Table 1 summarizes the demographic and clinical characteristics of the participants. The numbers of manifestations of the subjects is presented in Additional file 5. The structure of the LTM model is shown in Fig. 1. The latent variables are labeled with the capital letter “Y.” The numbers in parentheses represent the possible number of states of the latent variable. In our sample, all latent variables had two possible states; participants were divided between these two states and each state represented a cluster of participants. The meaning of each state was determined by considering the probability distributions in that state of the symptom variables directly connected to the latent variable. The latent variables revealed either clinical feature co-occurrence patterns or clinical feature mutual-exclusion patterns. The strengths of clinical feature dependencies in each latent variable are visually depicted by the width of the bars. For example, Y18 (located at the right-hand lower corner) was strongly correlated with “thirst,” moderately correlated with ‘‘favour of drinking” and weakly correlated with “pale and large amount of urine.”

**Table 1:** Demographic and clinical characteristics

Variables^a^	Total (n = 142)
Age, years	49.5 ± 9.7
Sex, male/female	29/113
Education attainment, years	10.7 ± 3.2
Marital status	
Never married	24 (16.9)
Married/cohabiting	85 (59.9)
Divorced/widowed	33 (23.2)
Occupation	
Professional and associate professional	6 (4.2)
Skilled and semi-skilled worker	20 (14.1)
Unskilled worker	16 (11.3)
Retired	24 (16.9)
Unemployed/housework	76 (53.5)
Chronic medical illnesses^b^	34 (23.9)
Insomnia duration, years	10.3 ± 9.5
Depression duration, years	8.6 ± 11.6
Insomnia severity index score	19.9 ± 3.2
Hamilton depression rating scale-17 item total score	10.5 ± 4.2

^a^Data are presented as mean ± SD or number (%)

^b^Participants were on regular medications for their medical illnesses

### Clinical feature co-occurrence patterns

Table 2 shows the conditional probability distribution of each clinical feature with co-occurrence patterns. Each latent variable represents a division of the participants, and each state of the latent variable denotes a cluster. For example, the latent variable Y18 had two states, denoted as Y18 = s0 and Y18 = s1. It represented a division of the participants into two clusters. Y18 formed a latent class model with three clinical feature variables (Fig. 1). Table 2 shows the clinical feature variables that contributed significant cumulative information coverage (at least 95 %). The cumulative information coverage of a variable in the ordered list is a ratio, in which the numerator is the amount of information about the partition contained in the variable and all the variables before it, and the denominator is the amount of information about the partition contained in all the variables. For Y18, the coverage reached 95 % at the clinical features “thirst” and “desire to drink.” In this case, we concluded that the clinical features “thirst” and “desire to drink” were sufficient to characterize the differences between the two clusters. The remaining clinical feature “pale and large amount of urine” was ignored and not included in Table 2. Therefore, only the probability distributions of the two clinical feature variables “thirst” and “favor of drinking” in the two clusters Y18 = s0 and Y18 = s1 are presented (Table 2).

**Table 2:** Comparison between latent variables and existing CM syndromes

Latent variables^a^			CM syndromes									
			1	2	3	4	5	6	7	8	9	10
Y18	Y18 = s0	Y18 = s1										
	0.43	0.57										
Thirst	0.07	1	●									
Favour of drinking	0.18	0.78	●									
Y17	Y17 = s0	Y17 = s1										
	0.34	0.66										
Dry mouth	0.39	1			●	●			●		●	
Dry throat	0.01	0.53			●	●			●		●	
Bitter taste	0.19	0.64	●	●	●				●			
Agitation	0.33	0.66				●						
Y16	Y16 = s0	Y16 = s1										
	0.62	0.38										
Weary limbs	0.31	1						●				
Sore knees	0.21	0.83				●			●		●	
Hypochondriac pain	0.01	0.29	●		●							
Backache	0.51	0.88			●	●			●		●	
Pain in the chest and hypochondrium	0	0.15	●									
Aphthous stomatitis	0.2	0.53			●						●	
Y15	Y15 = s0	Y15 = s1										
	0.81	0.19										
Heavy headedness	0.18	0.88		●								
Head distension	0.25	0.91	●									
Difficulty falling asleep alone	0.02	0.25								●		
Y14	Y14 = s0	Y14 = s1										
	0.69	0.31										
Insomnia with vexation	0.05	0.64				●			●		●	
Restless sleep	0.48	0.93		●		●					●	
Vexation in sitting and lying down	0	0.21								●		
Difficulty staying asleep	0.79	1				●			●	●	●	
Y13	Y13 = s0	Y13 = s1										
	0.82	0.18										
Sleeping late at night	0.29	1			●	●					●	
Frequent awakening with a start	0.14	0.75								●		●
Half asleep	0.35	0.94						●				
Poor memory	0.93	0.66			●			●	●		●	
Y12	Y12 = s0	Y12 = s1										
	0.48	0.52										
Headache	0.25	0.82	●	●	●							
Excessive dreaming	0.38	0.91	●		●			●	●	●	●	●
Night sweating	0.06	0.55			●	●					●	
Feverish sensations in the palms soles and chest	0.02	0.43			●	●			●		●	
Tinnitus	0.18	0.54	●		●	●			●		●	
Y11	Y11 = s0	Y11 = s1										
	0.88	0.12										
Nausea	0.01	1		●								
Poor appetite	0.16	0.82	●	●		●		●	●			
Y10	Y10 = s0	Y10 = s1										
	0.61	0.39										
Fright palpitation	0.03	0.41									●	
Dyspnea	0.15	0.58								●		●
Tasteless	0.01	0.27						●				
Lassitude	0.78	1					●					
Oppression in the chest	0.18	0.53	●	●		●						●
Cold extremities	0.23	0.59									●	
Y09	Y09 = s0	Y09 = s1										
	0.46	0.54										
Frequent sighing	0.09	0.73	●									
Fatigue	0.27	0.81	●		●							
Vexation	0.14	0.64	●	●	●	●			●	●	●	
Unrefreshing sleep	0.72	0.99				●						
Dizziness	0.09	0.46	●	●	●	●		●	●		●	
Difficulty falling asleep with vexation	0.23	0.63				●					●	
Palpitation	0.06	0.34			●			●	●	●	●	●
Y08	Y08 = s0	Y08 = s1										
	0.39	0.61										
Thoughtful	0.34	1								●		
Y07	Y07 = s0	Y07 = s1										
	0.39	0.61										
Impatience	0.09	0.92	●		●						●	
Irritability	0	0.71	●		●						●	
Y05	Y05 = s0	Y05 = s1										
	0.55	0.45										
Belching	0.25	0.96		●		●	●					
Gastric stuffiness	0.03	0.42		●		●						●
Stuffiness and pain in stomach and abdomen	0.06	0.38				●						
Stuffiness in stomach and abdomen	0.13	0.48				●						
Profuse sputum	0.12	0.43		●								
Difficulty falling asleep	0.68	0.93	●		●	●		●	●	●	●	
Hypochondriac distension	0.02	0.17	●									
Y04	Y04 = s0	Y04 = s1										
	0.76	0.24										
Acid regurgitation	0.15	1		●					●			
Insomnia	0.56	1		●					●			

CM syndrome 1–10 refers to *Liver*-*qi stagnation transforming into fire*, *Internal disturbance of phlegm*-*heat*, *Liver fire flaming upward*, *Stomach disharmony*, *Stomach qi disharmony*, *Deficiency of both the heart and spleen*, *Hyperactivity of fire due to yin deficiency*, *Qi deficiency of the heart and gallbladder*, *Heart*-*kidney noninteraction*, and *Heart deficiency with timidity*, respectively. The dots represent that the clinical features are often present in the CM syndromes

^a^The latent variables are presented starting from the bottom of the latent tree model

The two clinical features “thirst” and “favor of drinking” did not always co-occur in the first cluster, but they co-occurred with high probabilities (1.00 and 0.78, respectively) in the second cluster. The probability distribution indicates that these two clinical features co-occurred in the cluster Y18 = s1. As the co-occurrence is probabilistic in nature, it is called a probabilistic clinical feature co-occurrence pattern. This co-occurrence pattern was compared with the 10 most commonly used CM syndromes identified by the previous systematic review of CM syndromes associated with insomnia [21] to interpret the CM syndromes indicated by the latent variables. The presence of the probabilistic pattern Y18 = s1, including “thirst” and “desire to drink,” suggested the syndrome *Liver qi stagnation transforming into fire* (*ganyu huahuo*) [21].

The latent state Y17 = s1 captured the probabilistic co-occurrence of “dry mouth,” “dry throat,” “bitter taste,” and “agitation” This pattern was present in 66 % of the participants. According to our previous systematic review, these four clinical features co-occur in many CM syndromes. In fact, when interpreting a clinical feature co-occurrence pattern, it is necessary to interpret a pattern as a whole, rather than as individual clinical features. The single clinical feature “bitter taste” could be explained by the CM syndromes *Liver qi stagnation transforming into fire; Internal disturbance of phlegm*-*heat* (*tanre neirao*); *Liver fire flaming upward* (*ganhuo shangyan*); and *Hyperactivity of fire due to yin deficiency* (*yinxu huowang*). However, the CM syndromes *Liver qi stagnation transforming into fire* and *Internal disturbance of phlegm*-*heat* could not explain the other clinical features, such as dry mouth and dry throat, and hence they are not appropriate interpretations. A more appropriate explanation of Y17 = s1 would be *Liver fire flaming upward, Stomach disharmony*, and *Hyperactivity of fire due to yin deficiency*, because these syndromes explain the co-occurrence of most of the clinical features.

The distributions for the cluster Y16 = s1 indicated that “weary limbs” co-occurred with “sore knees,” “hypochondriac pain,” “backache,” “pain in the chest and hypochondrium,” and “aphthous stomatitis.” According to our systematic review, no CM syndromes contained all these clinical features. In this case, a total co-occurrence pattern could not be interpreted because the clinical feature variables under Y16 did not directly match any particular CM syndrome. Therefore, we attempted to break this cluster into three parts. The feature “weary limbs” (*tui kun*) was present in the CM syndrome *Deficiency of both the heart and spleen* (*xinpi liangxu*) and it occurred in 100 % of the participants in the cluster Y16 = s1. “Aphthous stomatitis” is only present in the CM syndrome *Liver fire flaming upward*, while *Heart*–*kidney noninteraction* (*xinshen bujiao*) contains three of the remaining four co-occurrence clinical feature variables.

The latent state Y15 = s1 captured the probabilistic co-occurrence of “heavy headedness,” “head distension,” and “difficulty falling asleep alone.” This pattern was only present in 19 % of the participants. This co-occurrence pattern did not belong to any of the CM syndromes identified in the previous systematic review [21]. Therefore, these clinical features were separately described as *Internal disturbance of phlegm*-*heat, Liver qi stagnation transforming into fire*, and *Qi deficiency of the heart and gallbladder* (*xindan qixu*).

The distribution of the cluster Y14 = s1, which represented 31 % of the participants, indicates that “difficulty staying asleep” co-occurred with “insomnia with vexation,” “restless sleep,” and “vexation in sitting and lying down.” In fact, no CM syndromes contain all these clinical features. “Vexation in sitting and lying down” is observed in *Qi deficiency of the heart and gallbladder.* The other three clinical features could be explained by the CM syndromes *Stomach disharmony* (*weifu shihe*) and *Heart*–*kidney noninteraction* (*xinshen bujiao*).

The latent state Y13 = s1 captured the probabilistic co-occurrence of “sleeping late at night” (experienced by 100 % of the participants) with “frequent awakening with a start,” “half asleep,” and “poor memory.” The co-occurrence pattern of these four clinical features did not match any particular CM syndrome [21]. The co-occurrence pattern of “half asleep” and “poor memory” might be explained by *Deficiency of both the heart and spleen* (*xinpi liangxu*) and the co-occurrence pattern of “sleeping late at night” and “poor memory” may be explained by *Liver fire flaming upward* and *Heart*–*kidney noninteraction*.

The latent state Y12 = s1 captured the probabilistic co-occurrence of five clinical features, including “headache,” “excessive dreaming,” “night sweating,” “feverish sensations in the palms, soles, and chest,” and “tinnitus.” The co-occurrence pattern of these five clinical features is described in the CM syndrome *Liver fire flaming upward*.

The latent state Y11 = s1 captured the probabilistic co-occurrence of “nausea” and “poor appetite.” “Poor appetite” is included in five of the 10 CM syndromes commonly associated with insomnia; however, only the CM syndrome *Internal disturbance of phlegm*-*heat* (*tanre neirao*) can explain the co-occurrence of “nausea” and “poor appetite.”

The distributions for the cluster Y10 = s1 indicated that “lassitude” tended to co-occur with “fright palpitations,” “dyspnea,” “loss of taste,” “oppression in the chest,” and “cold extremities.” However, this co-occurrence pattern is not described in any of the CM syndromes commonly associated with insomnia [21]. Only *Heart*–*kidney noninteraction* and *Heart deficiency with timidity* include two of these six co-occurrence clinical features.

The latent state Y09 = s1 captured the probabilistic co-occurrence of seven clinical features: “frequent sighing,” “fatigue,” “vexation,” “unrefreshing sleep,” “dizziness,” “difficulty falling asleep with vexation,” and “palpitations.” None of the commonly used CM syndromes had such a co-occurrence pattern. “Frequent sighing” and “unrefreshing sleep” are present in the CM syndromes *Liver qi stagnation transforming into fire* and *Stomach disharmony*, respectively; the remaining clinical features could be explained by *Liver fire flaming upward* and *Heart*–*kidney noninteraction*.

The latent variable Y08 had only one clinical feature variable, “thoughtful,” which was compatible with *Qi deficiency of the heart and gallbladder*. The latent state Y07 = s1 captured the probabilistic co-occurrence of “impatience” and “irritability.” This pattern was present in 61 % of the participants and suggested *Liver qi stagnation transforming into fire, Liver fire flaming upward*, and *Heart*–*kidney noninteraction*.

The latent state Y05 = s1 captured the probabilistic co-occurrence of seven clinical features: “belching,” “gastric stuffiness,” “stuffiness and pain in stomach and abdomen,” “stuffiness in stomach and abdomen,” “profuse sputum,” “difficulty falling asleep,” and “hypochondriac distension.” None of the commonly used CM syndromes describe the co-occurrence of these seven clinical features, but the CM syndrome *Stomach disharmony* includes five of the seven clinical features.

The latent state Y04 = s1 captured the probabilistic co-occurrence of “acid regurgitation” and “insomnia.” This pattern was present in 24 % of the participants. The co-occurrence pattern was compatible with the CM syndromes *Internal disturbance of phlegm*-*heat* and *Hyperactivity of fire due to yin deficiency*.

### Clinical feature mutual-exclusion patterns

Table 3 presents the latent variables that have mutual-exclusion patterns. The first cluster (Y06 = s0) contained 31 % of the participants and the second cluster (Y06 = s1) contained 69 % of the participants. The Y06 = s0 cluster indicated that when “string-like pulse” is detected, “fine pulse,” “sunken pulse,” and “slippery pulse” tended not to occur, while in Y06 = s1, “fine pulse,” “sunken pulse,” and “slippery pulse” tended to co-occur.

**Table 3:** Latent variables with mutual-exclusion patterns

Latent variables		
Partition given by Y06	Y06 = s0	Y06 = s1
	(0.31)	(0.69)
String like pulse	1.00	0.32
Fine pulse	0.27	0.78
Sunken pulse	0.08	0.39
Slippery pulse	0.44	0.78
	Y03 = s0	Y03 = s1
Partition given by Y03	(0.79)	(0.21)
White coating	0.63	0.00
Yellow coating	0.14	0.70
Partition given by Y02	Y02 = s0	Y02 = s1
	(0.54)	(0.46)
Pale tongue	1.00	0.00
Red tongue	0.04	0.68
Partition given by Y01	Y01 = s0	Y01 = s1
	(0.48)	(0.52)
Thin coating	1.00	0.05
Slimy coating	0.00	0.23

Y03 revealed that “white coating” and “yellow coating” were often mutually exclusive. “White coating” was not present in Y03 = s1, but the probabilistic occurrence of “yellow coating” was 0.70. However, in Y03 = s0, the tongue features co-occurred in a small percentage of participants (white coating = 0.63, yellow coating = 0.14). Y02 indicated that “pale tongue” and “red tongue” were mutually exclusive. In Y02 = s0, when “pale tongue” occurred, only 4 % of participants presented with a “red tongue,” whereas in Y02 = s1, when participants had a “red tongue,” none of the participants presented with a “pale tongue.”

Our analysis showed that the first cluster (Y01 = s0) consisted of 48 % of the participants and the second cluster (Y01 = s1) consisted of 52 % of the participants. In Y01 = s0, “thin coating” often occurred (1.00), while “slimy coating” was absent (0.00); whereas in Y01 = s1, “thin coating” and “slimy coating” tended to co-occur, although with low probabilities (0.05 and 0.23, respectively).

Overall, we identified many probabilistic clinical feature co-occurrences and mutual-exclusion patterns in a sample of participants with insomnia and a history of major depression using LTA. Some of the clinical feature co-occurrence patterns could be explained by CM syndromes; these are summarized in Table 4.

**Table 4:** Summary of co-occurrence patterns that have CM syndrome connotations

Latent states	Co-occurrence patterns	Relevant CM syndromes
Y18 = s1	Thirst, favor of drinking	*Liver*-*qi stagnation transforming into fire*
Y17 = s1	Dry mouth, dry throat, bitter taste, agitation	*Liver fire flaming upward, Stomach disharmony* *Hyperactivity of fire due to yin deficiency*
Y16 = s1	Weary limbs, sore knees, hypochondriac pain, backache, pain in the chest and hypochondrium, aphthous stomatitis	*Deficiency of both the heart and spleen* *Liver fire flaming upward* *Heart*-*kidney noninteraction*
Y15 = s1	Heavy headedness, head distension, difficulty falling asleep alone	*Internal disturbance of phlegm*-*heat* *Liver*-*qi stagnation transforming into fire* *Qi deficiency of the heart and gallbladder*
Y14 = s1	Insomnia with vexation, vexation, restless sleep, vexation in sitting and lying down	*Qi deficiency of the heart and gallbladder* *Stomach disharmony* *Heart*-*kidney noninteraction.*
Y13 = s1	Sleeping late at night, frequent awakening with a start, half asleep, poor memory	*Liver fire flaming upward* *Deficiency of both the heart and spleen* *Heart*-*kidney noninteraction*
Y12 = s1	Headache, excessive dreaming, night sweating, feverish sensations in the palms, soles and chest, tinnitus.	*Liver fire flaming upward*
Y11 = s1	Nausea, poor appetite	*Internal disturbance of phlegm*-*heat*
Y10 = s1	Lassitude, fright palpitation, dyspnea, tasteless, oppression in the chest, cold extremities	*Heart*-*kidney noninteraction,* *Heart deficiency with timidity*
Y09 = s1	Frequent sighing, fatigue, vexation, unrefreshing sleep, dizziness, difficulty falling asleep with vexation, palpitation.	*Liver*-*qi stagnation transforming into fire* *Stomach disharmony* *Liver fire flaming upward* *Heart*-*kidney noninteraction*
Y08 = s1	Thoughtful	*Qi deficiency of the heart and gallbladder*
Y07 = s1	Impatience, irritability	*Liver*-*qi stagnation transforming into fire* *Liver fire flaming upward* *Heart*-*kidney noninteraction*
Y05 = s1	Belching, gastric stuffiness, stuffiness and pain in stomach and abdomen, stuffiness in stomach and abdomen, profuse sputum, difficulty falling asleep, hypochondriac distension	*Stomach disharmony*
Y04 = s1	Acid regurgitation, insomnia	*Internal disturbance of phlegm*-*heat* *Hyperactivity of fire due to yin deficiency*

## Discussion

Insomnia diagnostic criteria in modern Western medicine have focused entirely on insomnia symptoms and the functional impairments caused by insomnia [26]. The present study is based on a 93-item standardized Chinese Medicine Insomnia Symptom Checklist (Additional file 5) containing symptoms and signs, mostly non-sleep-related, that are of interest from a CM perspective. Our LTA of these clinical feature data revealed 18 latent variables. The clinical feature co-occurrence patterns captured by the latent variables indicated CM syndromes. In addition, the LTA also revealed the proportion of participants who presented such co-occurrence patterns. There were six clusters representing more than 50 % of the sample: Y18 = s1, Y17 = s1, Y12 = s1, Y09 = s1, Y08 = s1, and Y07 = s1. The clinical feature co-occurrence patterns identified in these clusters could be explained by the CM syndromes of *Liver qi stagnation transforming into fire*, *Liver fire flaming upward*, *Stomach disharmony*, *Hyperactivity of fire due to yin deficiency*, *Heart*–*kidney noninteraction*, and *Qi deficiency of the heart and gallbladder.* Our previous systematic review [21] identified these five CM syndromes as common patterns found in insomnia participants.

Our LTA revealed 18 latent variables, of which 13 captured probabilistic clinical feature co-occurrence patterns, four captured clinical feature mutual-exclusion patterns, and one connected with a single clinical feature variable. These latent variables matched our understanding of CM syndromes, suggesting that the co-occurrence patterns identified from a sample of participants with insomnia and a history of major depression indicate CM syndromes. The latent variables could be used to construct a CM syndrome differentiation algorithm by performing a joint clustering analysis using the latent variables that are related to the same CM syndrome. In joint clustering, latent class analysis is performed on the participant population using selected latent variables as features. The analysis divides the participants into two or more clusters, and the participant clusters correspond to various syndrome types depending on which latent variables are selected for the analysis [27]. Future studies could be performed to achieve an expert consensus in selecting latent variables related to the same CM syndrome; these could then be put into a joint clustering analysis.

The LTA also identified four latent variables that captured clinical feature mutual-exclusion patterns. These variables were related to tongue and pulse features. In Y06, “string-like pulse” tended not to co-occur with “fine pulse,” “sunken pulse,” and “slippery pulse.” However, the mutual-exclusion pattern was not absolute. In CM theory, these four pulse features could present in different combinations. For example, someone with *Stomach disharmony* might present with a string-like and slippery pulse; a string-like and sunken pulse or a string-like and fine pulse could also be present in participants suffering from *cold*-*evil*. In Y01, thin tongue coating and slimy tongue coating were mutually exclusive, which matches CM theory: a thin coating represents an *evil* that has not invaded the body while a slimy coating represents the presence of *damp*-*evil* in the body, so these two tongue features were less likely to co-occur. The other two latent variables with mutual-exclusion patterns were related to the color of the tongue coating (Y03) and the color of the tongue body (Y02). The color of the coating tends to be either white or yellow (but not both), and the color of the tongue tends to be either pale or red (but not both).

There are few studies examining the benefit of CM syndrome differentiation. Further study would be useful to determine whether CM syndrome differentiation is of clinical significance.

Previous studies have attempted to examine CM syndromes using factor analysis and cluster analysis [26–29]. However, these statistical approaches cannot capture the nature of CM syndromes. For example, factor analysis assumes that the observed variables are a linear combination of the latent variables (CM syndromes) and that the factors are independent of each other; however, CM syndromes might be caused by inter-related factors [28]. Cluster analysis has previously been used to group clinical feature variables [29, 30] and to group participants [31]. In the former case, clinical feature variable clusters were interpreted as syndrome types. However, clinical feature variables that were similar (and hence grouped together) did not necessarily indicate the co-occurrence of clinical features or symptoms [29, 30]. In the latter case, participants were divided into mutually exclusive clusters [31]. This was also problematic because CM syndrome types can overlap.

Zhao et al. [14] identified 29 latent variables using LTA in a sample of participants with depression. The authors found that symptoms of *Liver qi stagnation* and *Yin deficiency* formed co-occurring patterns. The present study identified 18 latent variables; these were associated with *Liver fire flaming upward, Stomach disharmony*, and *Heart*–*kidney noninteraction*. The difference in the number and CM indications of the latent variables identified might be because of differences in sample characteristics. Although the present study and that of Zhao et al. [14] included participants with depression, our participants’ depression was mostly in remission (Hamilton Depression Rating Scale score ≤18) although they had persistent insomnia symptoms, while Zhao’s study included participants with a diagnosis of depression regardless of severity.

There were some limitations in our study. First, our sample consisted of a specific group of participants in full or partial remission from major depressive disorder with insomnia, and hence the results might not generalize to other insomnia samples. Second, our Chinese Medicine Insomnia Symptom Checklist only covered clinical features of the previous 2 weeks, regardless of their frequency and severity. However, given that the frequency and severity criteria of clinical features have not been clearly defined in textbooks and previous reviews, the inclusion of these factors may have complicated the model. Third, we compared our findings with a systematic review [21] of CM syndromes commonly associated with insomnia to describe the CM syndrome indications of the LTA results. However, our LTA results have not been replicated or validated against CM practitioners’ observations in clinical practice nor compared with models using other statistical methods, such as factor analysis and cluster analysis. Finally, although our standardized Chinese Medicine Insomnia Symptom Checklist included 93 items, some rare symptoms and signs, and pulse and tongue features, were inevitably not included. Similarly, our interpretation of the latent variables was based on the 10 most commonly used CM patterns identified in a systematic review [21]; hence, some rare CM patterns were not compared.

## Conclusion

Our analysis using LTMs in a sample of depressed subjects with insomnia revealed 13 clinical feature co-occurrence patterns, four mutual-exclusion patterns, and one with a single clinical feature variable.

## Additional files

10.1186/s13020-016-0076-y Research ethic approval.
10.1186/s13020-016-0076-y Research protocol.
10.1186/s13020-016-0076-y Patient informed consent form.
10.1186/s13020-016-0076-y Chinese Medicine Insomnia Symptom Checklist.
10.1186/s13020-016-0076-y Numbers (percentages) of manifestations of the subjects (N = 142).

## Abbreviations

DSM-IV-TR: diagnostic and statistical manual of mental disorders, fourth edition, text revision edition
LCM: latent class model
LTM: latent tree model
CM: Chinese medicine
