# Supplementary material for: Identification of Chinese medicine syndromes in persistent insomnia associated with major depressive disorder: a latent tree analysis
Source: Chin Med. 2016 Feb 12;11:4. doi: 10.1186/s13020-016-0076-y (PMC4751631; doi:10.1186/s13020-016-0076-y)
Supplement: Supplementary file 2 — 10.1186/s13020-016-0076-y Research protocol. [file 13020_2016_76_MOESM2_ESM.doc]

**PROTOCOL SYNOPSIS**

**TITLE:**

A randomized controlled trial of acupuncture for residual insomnia associated with major depressive disorder

**OBJECTIVE:**

To evaluate the efficacy and safety of acupuncture treatment for residual insomnia and other residual symptoms associated with major depressive disorder

**TRIAL DESIGN:**

Randomized controlled trial

**TRIAL POPULATION:**

This trial will recruit 96 subjects who fulfill the inclusion/ exclusion criteria.

**CLINICAL EFFICACY VARIABLES:**

Primary outcomes measures are sleep parameters measured by actigraphy, Insomnia Severity Index (ISI) and sleep log, and depressive state as measured by Hamilton Depression Rating Scale (HDRS). Secondary outcomes are Pittsburgh Sleep Quality Index (PSQI) and Sheehan Disability Index (SDI).

**Introduction**

Major depressive disorder (MDD) is a debilitating illness that affects an estimated 13% of the general population.^1^ It was the fourth leading cause of disability and disease, accounting for 4.4% of the total disability-adjusted life years in the year 2000.^2^ Residual symptoms in MDD can be defined as core depressive symptoms that have not resolved with treatment.^3^ Insomnia is one of the most common residual symptoms associated with MDD.

Previous studies have shown that residual symptoms associated with MDD are common. Approximately one third of patients with MDD fail to fully respond to antidepressant treatment of adequate dose and duration.^4^ In one study, Nierenberg et al. investigated 108 depressed patients who had received an 8-week treatment of fluoxetine, an antidepressant.^5^ They found that the prevalence of residual insomnia was 44%, followed by fatigue (38%) and diminished interest (27%). Nelson et al. showed that the prevalence of different subtypes of residual insomnia was: difficulty initiating asleep (37%), midnight awakening (33%), and early morning awakening (20%).^6^

Effective treatment of residual symptoms is essential, since their persistence correlates with a greater risk of MDD recurrence and relapse as well as worse psychosocial functioning. Tranter et al. found that depressed patients with residual symptoms had a relapse rate 3-6 times higher than in those who experienced full remission.^7^ In another study, Mojtabai et al. found that the severity of residual symptoms was associated with the number of days of impaired occupational functioning.^8^

Residual insomnia can be a symptom of MDD that requires longer time to remit, a side effect of pharmacological treatment, or a feature etiologically different from but coexist with MDD.^3^ In a study that compared the rate of residual insomnia following pharmacotherapy or cognitive behavioral therapy for MDD. Carney et al. found that the rate of residual insomnia after drug and non-drug treatment was 53% and 50%, respectively, suggesting that medications alone cannot account for the residual insomnia symptoms.^9^

There have been limited studies examining the treatment of residual insomnia in MDD. One approach is to treat residual insomnia by augmentation of antidepressant treatment using drug and non-drug approaches. On the other hand, pharmacological or psychological treatments targeting insomnia symptoms may enhance the recovery of both sleep and mood disturbance.

Medications that are used to treat insomnia are limited by adverse effects, abuse and dependence. Psychological treatments are time intensive and require significant training for effective implementation. Pharmacological and psychological treatments of MDD have similar limitations. Acupuncture is one of the safest complementary therapies and has been used to treat insomnia and depression.^10,11^ Our previous randomized placebo-controlled study has shown that electroacupuncture is effective for primary insomnia.^12^

We aim to extend the use of acupuncture for treating residual insomnia associated with MDD. The reason for choosing this patient population is that the currently available pharmacological and psychological treatments have their limitations and may not be effective. In addition, the outcome and functioning of partially remitted depressed patients would be enhanced with remission of the residual symptoms.

We have conducted a pilot study comparing the efficacy of electroacupuncture, minimal acupuncture and placebo acupuncture for the treatment of residual insomnia in 30 subjects with MDD. We found that the subjects in electroacupuncture and minimal acupuncture groups had significantly greater improvement in Insomnia Severity Index (ISI) and Pittsburgh Sleep Quality Index (PSQI) scores at 4-week posttreatment than those in placebo acupuncture group (ANCOVA, ISI: F = 5.33, p = 0.01; PSQI: F = 7.93, p = 0.002). In view of the initial positive findings, it is important to extend the study for a longer follow-up period, in a larger sample and using objective outcome measure.

**Aims**

To evaluate efficacy and safety of electroacupuncture for residual insomnia associated with MDD.

**Plan of Investigation**

*Study Design*

The proposed study is a randomized, prospective, placebo-controlled, single-blind trial of electroacupuncture for residual insomnia associated with MDD. The study design aims to differentiate three plausible mechanisms for sleep improvement in patients with insomnia symptoms: (1) needling at specific sites based on traditional Chinese medicine (TCM) theory; (2) characteristic and incidental elements of acupuncture treatment, e.g. credibility of intervention, patient expectations;^13^ and (3) underlying fluctuations in the disorder, regression to the mean, or non-specific improvement associated with participating in a clinical trial.

Eligible patients will be randomized in a ratio of 1:1:1 to: (1) traditional electroacupuncture; (2) placebo acupuncture using Streitberger needle; and (3) waiting list control group. Active or placebo acupuncture treatment will be given 3 sessions per week for 3 weeks. Patients in the waiting list control group will not receive any treatment for a period of 8 weeks after randomization. After that time, they will receive the same treatment as in the traditional electroacupuncture group. The subjects’ sleep and residual symptoms will be assessed at baseline, after completing 2 weeks of acupuncture treatment, and at 1-week, 5-week and 13-week posttreatment.

*Subjects*

Outpatients, aged from 18 to 65 years, with a previous DSM-IV MDD and a chief complaint of insomnia will be invited to participate in this study. Written informed consent would be obtained prior to study procedure. Subjects would be excluded if they: (1) have a Hamilton Depression Rating Scale scores above 18; (2) have sleep apnea or periodic limbs movement disorder detected by overnight polysomnography; (3) have suicidal risk; (4) have previous history of schizophrenia, other psychotic disorders, and bipolar disorder; (5) are pregnant, breast-feeding, or woman of childbearing potential not using adequate contraception; (6) have infection or abscess close to the site of selected acupoints and in the investigator’s opinion inclusion is unsafe; and (7) are taking Chinese herbal medicine or over-the-counter drugs which are intending for insomnia.

The number of subjects to be recruited in each treatment group would be 30.

*Treatment Protocol*

Traditional Acupuncture

Patients will be treated at bilateral Ear Shenmen (神門), Ear point Heart (心), Sishencong EX-HN1 (四神聰), Anmian (安眠), and unilateral Yingtong EX-HN3 (印堂) and Baihui DU20 (百會). The acupoints are empirical for treating insomnia in term of TCM theory and have been used in our previous study of acupuncture for primary insomnia.

Skin around these points will be sterilized by 75% alcohol and then sterilized disposable needle is inserted to each point by a registered Chinese medicine practitioner. “De qi”(an irradiating feeling considered to be indicative of effective needling) is achieved if possible. An electric-stimulator (CEFAR Acus II, Lund, Sweden) is connected to these needles to give an electric-stimulation in continuous wave, frequency of 4 Hz, 0.45 ms square wave pulses and constant current. Surgical tape or hair pin will be adhered to the needles. It is to increase the similarity between treatment and control group which will be described later. The needles will be left for 30 min and then removed. Acupuncture treatment will consist of three sessions per week for 3 consecutive weeks.

Placebo Acupuncture

The sterilization procedure is the same as the acupuncture group for insomnia. Placebo needles designed by Streitberger will be used.^15^ The placebo needles are blunt needle that will not penetrate the skin during needle insertion. The handles of these placebo needles will slide over the needle when it is compressed, giving it the appearance of penetrating the skin. The placebo needles are inserted to the site 1 inch beside the acupoints in order to avoid the acupressure effect. The needles are held by a surgical tape or hair pin in hairy region to imitate the retention of needles. The needles are connected to an electric-stimulator with zero frequency and amplitude. The number, duration and frequency of the treatment sessions, and the intervention procedure will be the same for traditional acupuncture and placebo acupuncture. To ensure standardization of the acupuncture techniques, all acupuncture will be performed by the same registered Chinese medicine practitioner. The patients will be treated in separated room or time to prevent communication between treatment group and placebo acupuncture group.

Method of randomization

The subjects will receive the Structured Clinical Interview for DSM-IV to evaluate psychiatric diagnoses and polysomnography to exclude specific sleep disorders prior to randomization. Block randomization is used in the randomization procedure. This is performed by using Excel to generate a randomized block list.^16^ Subjects are randomly assigned to one of the three groups after baseline visit.

Method of blinding

The subjects are told as follows “This study is to evaluate the therapeutic effects of acupuncture on treating insomnia in depressive patients. You will be randomly assigned to traditional acupuncture group for insomnia or acupuncture-like placebo treatment group”; “Traditional acupuncture is an acupuncture treatment that conventionally used in Chinese medicine practice; placebo acupuncture is a procedure which mimics the real acupuncture procedure and give you a perception that you are receiving the acupuncture treatment”. The researchers who analyse the data will be blind to the treatment allocation.

Concomitant Therapy

Antidepressants will be maintained the same dosage during the study period. Sedatives, hypnotics or anxiolytics are allowed during the study. The subjects are told to record their dosage of these medications. Drug counts will be conducted at each study visit. Individual psychotherapy should be maintained if it is ongoing prior to the study. Any new form of therapy or education is disallowed.

*Outcome Assessment*

Subjective assessment

1. Insomnia Severity Index (ISI) (Primary outcome measure)

ISI is a 7-item self-rating scale. The subjects rate their severity of sleep problem and the daytime function performance due to insomnia using a 5-point Likert scale.

2. Sleep log

The subjects record their daily sleep-wake schedule on a standardized sleep log.

3. Hamilton Depression Rating Scale (HDRS)

HDRS is a 21-item scale which the clinicians used to rate the severity of patients’ depression. HDRS rates the severity of other depressive symptoms such as low mood, insomnia, agitation, anxiety and weight-loss.

4. Pittsburgh Sleep Quality Index (PSQI)

PSQI is a self-rating scale that assesses the subjects’ sleep in the past month. It has seven components, namely subjective sleep quality, sleep latency, sleep duration, habitual sleep efficiency, sleep disturbance, use of sleeping medication, and daytime dysfunction.

5 Sheehan Disability Index (SDI)

SDI is a 3-item self-rating scale that assesses the subjects’ functioning regarding work/study, social life and family.

Objective assessment

Actigraph

Actigraph is a watch-like device used to estimate sleep-wake schedules by measurement of activity. The subjects are asked to wear the actigraph on the dominant wrists prior to light off for 3 consecutive nights. Subjects will be told to have usual bedtime when they are measured by actigraph.

Assessment for safety

Reason for withdrawal

When a subject withdraws before completing the study, the reason for withdrawal will be recorded.

Adverse events

Adverse events will be assessed at each visit. The researcher will ask using open-ended questions whether the subjects experience any adverse events during the assessment visit.

Assessment for credibility

Credibility of treatment rating scale (modified by C Vincent) will be used to assess the credibility of the treatments.^17^ The 4-item scale is specially designed to assess the credibility of acupuncture.

*Data Analysis*

Sample size estimation

Insomnia Severity Index (ISI) is selected as the primary outcome measure. The sample size estimation is based on our pilot study. We found that the SD of ISI score in the treatment and control group was 4.7 and 3.6, respectively, while the baseline and endpoint ISI score correlation coefficient was roughly 0.5. Vickers and Altman proposed that analysis of covariance (ANCOVA) was the preferred approach to analyse controlled trials with baseline and follow up measurements.^18^ We estimate the sample size requirement per treatment group using:

$$n=\frac{(\delta_{1}^{2}+\delta_{2}^{2})[z_{\alpha/2}+z_{\beta}]^{2}}{\Delta^{2}}$$

(1- ρ^2^)

An adjustment factor (1- ρ^2^) is used to determine the sample size in each group;^19^ and ρ is the baseline and endpoint measure correlation coefficient.

Let type I error α = 0.05; type II error β = 0.20; and power (1-β) = 80%. The SD δ_1_= 4.7, δ_2_ = 3.6 and ρ = 0.5. A clinically significant treatment effect is taken as a 3 ISI point between-group difference (Δ = 3). Therefore, a sample size of 23 is needed in each group. We estimate a 20% attrition rate; hence a sample size of around 30 in each group (90 in total) is planned for this study.

Data Management and Analysis

All data are double-entered and checked for consistency prior to analysis.

Baseline differences between the electroacupuncture, minimal and placebo acupuncture groups would be examined using unpaired t-test or χ^2^ test. The last observation carried forward method would be used to handle missing data. The outcome measures during acupuncture treatment and at posttreatment would be compared with that at baseline using paired t-test. A 2 × 3 (time × group) ANCOVA with baseline measure as a covariate would be used to investigate differences between the electroacupuncture, minimal and placebo acupuncture groups. Standardized effect size would be computed by dividing the difference in means by the pooled standard deviation to estimate treatment impact. The clinical significance of the interventions would be estimated by the proportion of participants who reached sleep-diary SOL or WASO of 30 minutes or less and SE of at least 85%, and χ^2^ or Fisher exact test would be used to test for group differences.

Safety analyses

Subjects who receive at least 1 acupuncture treatment will be included for safety analyses. Adverse events will be coded using the WHO Adverse Reaction Terminology Dictionary. Special attention will be given to those subjects who have discontinued treatment due to an adverse event or who experience a severe or serious adverse event.

**Key References**

1. [Hasin DS, Goodwin RD, Stinson FS, Grant BF.](http://www.ncbi.nlm.nih.gov/pubmed/16203955?ordinalpos=32&itool=EntrezSystem2.PEntrez.Pubmed.Pubmed_ResultsPanel.Pubmed_DefaultReportPanel.Pubmed_RVDocSum) Epidemiology of major depressive disorder: results from the National Epidemiologic Survey on Alcoholism and Related Conditions. Arch Gen Psychiatry 2005;62:1097-1106.
2. [Ustün TB, Ayuso-Mateos JL, Chatterji S, Mathers C, Murray CJ.](http://www.ncbi.nlm.nih.gov/pubmed/15123501?ordinalpos=19&itool=EntrezSystem2.PEntrez.Pubmed.Pubmed_ResultsPanel.Pubmed_DefaultReportPanel.Pubmed_RVDocSum) Global burden of depressive disorders in the year 2000. Br J Psychiatry 2004;184:386-392.
3. Menza M, Marin H, Opper RS. Residual symptoms in depression: can treatment be symptom-specific? J Clin Psychiatry 2003;64:516-523.
4. Fava M, Davidson KG. Definition and epidemiology of treatment-resistant depression. Psychiatr Clin North Am 1996;19:179-200.
5. Nierenberg AA, Keefe BR, Leslie VC, et al. Residual symptoms in depressed patients who respond acutely to fluoxetine. J Clin Psychiatry 1999;60:221-225.
6. Nelson JC, Portera L, Leon AC. Residual symptoms in depressed patients after treatment with fluoxetine or reboxetine. J Clin Psychiatry 2005;66:1409-1414.
7. Tranter R, O'Donovan C, Chandarana P, Kennedy S. Prevalence and outcome of partial remission in depression. J Psychiatry Neurosci 2002;27:241-247.
8. Mojtabai R. Residual symptoms and impairment in major depression in the community. Am J Psychiatry 2001;158:1645-1651.
9. Carney CE, Segal ZV, Edinger JD, Krystal AD. A comparison of rates of residual insomnia symptoms following pharmacotherapy or cognitive-behavioral therapy for major depressive disorder. J Clin Psychiatry 2007;68:254-260.
10. Eisenberg DM, Davis RB, Ettner SL, et al. Trends in alternative medicine use in the United States, 1990-1997: results of a follow-up national survey. JAMA 1998;280:1569-1575.
11. Leo RJ, Ligot JS, Jr. A systematic review of randomized controlled trials of acupuncture in the treatment of depression. J Affect Disord 2007;97:13-22.
12. Yeung WF, Chung KF, Zhang SP, TG Yap, YP Chan. A Randomized Placebo-Controlled Trial of Acupuncture for Primary Insomnia. In: 12th Research Postgraduate Symposium, the University of Hong Kong, Li Ka Shing Faculty of Medicine. Hong Kong: the University of Hong Kong, Li Ka Shing Faculty of Medicine, 2007.
13. Paterson C, Dieppe P. Characteristic and incidental (placebo) effects in complex interventions such as acupuncture. BMJ 2005;330:1202-1205.
14. Melchart D, Streng A, Hoppe A, et al. Acupuncture in patients with tension-type headache: randomised controlled trial. BMJ 2005;331:376-382.
15. Streitberger K, Kleinhenz J. Introducing a placebo needle into acupuncture research. Lancet 1998;352:364-365.
16. Simon S. A simple approach for randomisation (17 Sep 1999). Retrieved 23 Oct 2008 from <http://bmj.bmjjournals.com/cgi/eletters/319/7211/703>
17. Vincent C. Credibility assessment in trials of acupuncture. Complementary Medical Research 1990;4:8-11.
18. Vickers AJ, Altman DG. Statistics notes: Analysing controlled trials with baseline and follow up measurements. BMJ 2001;323:1123-1124.
19. Borm GF, Fransen J, Lemmens WA. A simple sample size formula for analysis of covariance in randomized clinical trials. J Clin Epidemiol 2007;60:1234-1238.

**Table 1.** Study Assessment Schedule

| Assessment | Screening | Baseline | 2-week treatment completed | 1-week posttreatment | 5-week posttreatment | 13-week posttreatment |
| --- | --- | --- | --- | --- | --- | --- |
| Visit number | 1 | 2 | 3 | 4 | 5 | 6 |
| Day (D) | -21 to -14 D | -7 to 0 D | D15-D21 | D29 | D57 | D113 |
| Visit window (D) |  |  | ± 2 | ± 2 | ± 4 | ± 4 |
| Informed consent | √ |  |  |  |  |  |
| Review inclusion/ exclusion criteria | √ | √ | √ | √ | √ | √ |
| Medical & psychiatric history | √ |  |  |  |  |  |
| Vital signs | √ | √ | √ | √ | √ | √ |
| Demographics | √ |  |  |  |  |  |
| Physical examination | √ |  |  |  |  |  |
| Height & Weight | √ |  |  |  |  |  |
| SCID-I | √ |  |  |  |  |  |
| Wrist actigraphy  (3 days) |  | √ |  | √ | √ | √ |
| Overnight polysomnography | √ |  |  |  |  |  |
| Sleep Log (1 wk) |  | √ | √ | √ | √ | √ |
| ISI | √ | √ | √ | √ | √ | √ |
| HDRS | √ | √ | √ | √ | √ | √ |
| PSQI |  | √ |  | √ | √ | √ |
| SDI |  | √ | √ | √ | √ | √ |
| Credibility assessment |  |  | √ * |  |  |  |
| Recent & concomitant medication check | √ | √ | √ | √ | √ | √ |
| Adverse event monitoring | √ | √ | √ | √ | √ | √ |

Abbreviations: HDRS, Hamilton Depression Rating Scale; ISI, Insomnia Severity Index; PSQI, Pittsburgh Sleep Quality Index; SCID-I, Structured Clinical Interview for DSM-IV; SDI, Sheehan Disability Index.

* Credibility assessment is performed after the 2^nd^ and the 9^th^ (last) acupuncture treatment
