# Supplementary material for: Identification of Chinese medicine syndromes in persistent insomnia associated with major depressive disorder: a latent tree analysis
Source: Chin Med. 2016 Feb 12;11:4. doi: 10.1186/s13020-016-0076-y (PMC4751631; doi:10.1186/s13020-016-0076-y)
Supplement: Supplementary file 3 — 10.1186/s13020-016-0076-y Patient informed consent form. [file 13020_2016_76_MOESM3_ESM.docx]

PATIENT INFORMATION SHEET

STUDY TITLE: A Randomized Controlled Trial of Acupuncture for Residual Insomnia Associated with Major Depressive Disorder

Consent form version and date: VER 0.9 21/9/2008

Patient No:

Patient Initial:

You are invited to take part in a research study. Before you decide, it is important for you to understand why the research is being done and what it will involve. Please take time to read the following information carefully and discuss it with friends, relatives and your family doctor if you wish. Ask us if there is anything that is not clear or if you would like more information.

**PURPOSE OF THE STUDY**

The purpose of this study is to evaluate the clinical effectiveness of acupuncture treatment on residual insomnia in major depressive patients.

**TESTED INTERVENTION**

Traditional acupuncture will be tested in this study. Acupuncture has a long history and is widely adopted by Chinese medicine practitioners to treat different kinds of diseases, including insomnia.

**PARTICIPANT**

Individuals who have remitted from major depressive disorder with insomnia complaint are invited to participate in the study.

**STUDY PARTICIPATION**

It is up to you to decide whether or not to take part. If you do decide to take part you will be given this information sheet to keep and be asked to sign a consent form. If you decide to take part you are still free to withdraw at any time and without giving a reason. This will not affect the standard of care you receive and will not affect your relationship with Department of Psychiatry, Faculty of Medicine, University of Hong Kong or School of Chinese Medicine, Hong Kong Baptist University.

**STUDY DESIGN**

This is a randomized single-blinded controlled trial. We aim to recruit 90 subjects locally. You will be randomly assigned to one of the three groups. One is the traditional acupuncture treatment group, one is the acupuncture-like placebo group and the other one is waiting list control group; the investigators who carry out the assessment will not know in which treatment group you are.

Traditional acupuncture involves acupuncture needling at traditionally used acupoints according to Chinese medicine theory; acupuncture-like placebo group is a treatment that simulates the procedure of acupuncture treatment but may not have the effects of acupuncture. If you are assigned to waiting list control, you will not receive any treatment and under observation for 8-week. Participants will be put into groups and then compared. The chance of getting into each group is 1:1:1, i.e. equal chance.

You will be treated at the acupoints on head (bilateral Ear Shenmen (神門), Ear point Heart (心), Sishencong EX-HN1 (四神聰), Anmian (安眠), and unilateral Yingtong EX-HN3 (印堂) and Baihui DU20 (百會)). Electric-stimulation at a randomly selected dose will be delivered during the treatment to enhance therapeutic effects. The acupuncture needles are sterilized and disposable. The acupoints have been used in our previous studies of acupuncture for insomnia.

**STUDY PROCEDURE**

This study will last for 17 weeks. If you decide to take part in this study, you are required to come to this building have this visit for screening, 9 visits for a 30-45 min of receiving acupuncture treatment and 3 times for a 30 min of post-treatment assessment.

| **Week** | **0** | **1** | **2** | **3** | **4** | **9** | **17** |
| --- | --- | --- | --- | --- | --- | --- | --- |
| **Period** | Pretreatment | Treatment | | | 1 week after treatment | 5 weeks after treatment | 13 weeks after treatment |
| **Visit** | 1 visit for screening and 1 visit for PSG | 9 sessions of treatment | | | 1^st^ visit for assessment | 2^nd^ visit for assessment | 3^rd^ visit for assessment |

At the 1^st^ visit, you will be asked to complete a questionnaire and fill in a sleep log every day for one week. You will be asked in detail about your medical history and current medication. We will have a body check for you to collect your height, weight and heart rate at the 1^st^ visit for screening and the last visit for assessment. Besides, personal information such as date of birth, gender, education level, marital status, annual income, employment status will also be recorded at the 1^st^ visit.

If you fulfill the criteria of this study, the investigator will arrange a day for you to do an over-night polysomnography in Queen Mary Hospital. You are required to sleep overnight in Queen Mary Hospital for 1-night. The polysomnography can detect sleep disorders such as sleep apnea and periodic limbs movement disorder. If any of the above sleep disorder(s) is detected, you will be excluded from the study.

If no such sleep disorder is detected, the investigator will arrange the date of remaining visits for you. A watch-like device called actigraph will be used to record your sleep. You are asked to wear it for 3 consecutive nights and bring it back in the next visit. After the above procedure, you will be randomly assigned to traditional acupuncture treatment group, acupuncture-like placebo treatment group or waiting list control group.

If you are assigned to waiting list control group, you will not receive any treatment for 8-week. If you are assigned to traditional acupuncture treatment group or acupuncture-like placebo treatment group, you will receive the acupuncture treatment 3 times per week for 3 weeks, i.e. 9 visits of treatment. At the last treatment, you will be asked to wear the actigraph for 3 consecutive nights and fill in a sleep log everyday for 1 week. You will have assessments on your condition 5-weeks and 13-week after the acupuncture treatment.

**LIFE STYLE RESTRICTION**

You are required to stop any herbal remedies, over-the-counter medication or other acupuncture treatment, which may be intended for insomnia throughout the study. You are also required to have a usual bedtime during the days that wrist actigraph is used to record your sleep.

If you are potential for childbearing, you need to use adequate contraception. In case of pregnancy, you will be excluded from the study.

**ALTERNATIVES TREATMENT**

Conventional medications for insomnia are available.

**SIDE EFFECTS OF TAKING PART**

All medical interventions may have side effects.

Needle insertion will be slightly painful and lead to discomfort. Some patients may feel localized numbing during the treatment. However, all these discomfort feeling are transient and will not bring to serious sequela. Infection and hematoma at the acupuncture sites rarely occur. During the treatment, you should not move or using mobile phone. It will ensure the treatment efficacy and prevent accidents. If you feel any discomfort during the treatment, you should inform the researcher.

**DISADVANTAGES AND RISK OF TAKING PART**

It is not safe to give acupuncture to a pregnant woman; therefore pregnant women must not take part in this study. Women of childbearing age and sexually active must use an effective contraceptive method during the course of this study. Any woman who finds that she has become pregnant while taking part in the study should immediately tell the investigator.

**BENEFITS OF TAKING PART**

We hope that both treatments will improve your sleep problems and quality of life. However, this cannot be guaranteed. Moreover, the information we get from this study may help us to treat future depressed patients with insomnia better.

**NEW INFORMATION**

During the course of study, if there is any new information becomes available and which would affect your consent of taking part, the investigator will tell you.

**IF SOMETHING GOES WRONG**

Acupuncture is a safe treatment and has been used for over 2000 years in China. In Hong Kong, acupuncture treatment has been available in public and private Chinese medicine clinic for many years. The acupoints selected for use in this study have been used for treating insomnia by traditional Chinese medicine practitioners and tested in our previous study. Thus the acupuncture protocol in this study is safe. It is unlikely that event of serious physical injury resulting from your participation in this study. Be certain that you immediately notify the researcher if you have any adverse events. The investigator will decide whether you can continue with this study or not. This study does not have any indemnity arrangement for your injury/death.

**INFORMATION CONFIDENTIALITY**

All information which is collected about you during the course of the research will be kept strictly confidential. If you consent to take part in the research, any of your medical records may be inspected by the investigator for purpose of analyzing the results. They may also be looked at by people from regulatory authorities to check that the study is being carried out correctly. Your data will only be used for this study. The raw data will be stored for 10-year after completion of study and destroyed afterward.

**PRINCIPAL INVESTIGATOR**

This research is organized by Dr. Chung Ka Fai from Department of Psychiatry, Faculty of Medicine of the University of Hong Kong and Dr. Zhang Shi Ping from School of Chinese Medicine, Hong Kong Baptist University.

**ORGANIZATIONS WHICH HAVE REVIEWED THE STUDY**

The study has been reviewed and approved by the Institutional Review board of the University of Hong Kong/Hospital authority Hong Kong West Cluster and Committee on the Use of Human & Animal Subjects of Hong Kong Baptist University.

**CONTACT FOR FURTHER INFORMATION**

For further information or queries, please contact Dr. Chung Ka Fai at 2855 3067 or Dr. Zhang SP at 3411 2466.

If you feel you have not been treated according to the descriptions in this form, or your rights as a participant in research have been violated during the course of this project, you may contact the Institutional Review Board of the University of Hong Kong/Hospital Authority Hong Kong West Cluster at 2255 4086.

Thank you for taking part in the study and you will be given a copy of the information sheet and a signed consent form to keep.

PATIENT CONSENT FORM

Patient No:

Patient Initial:

STUDY TITLE: A Randomized Controlled Trial of Acupuncture for Residual Insomnia Associated with Major Depressive Disorder

Consent form version and date:

Name of Researcher: Dr. Chung Ka Fai

1. I confirm that I have read and understood the information sheet for the above study and have had the opportunity to ask questions.
2. I was informed with the nature of this clinical trial, my responsibility and the possible disadvantages brought by my voluntary of taking part in this study. I have received all the written related information of this study.
3. I understand that my participation is voluntary and that I am free to withdraw at any time, without giving any reason, with my medical care or legal rights being affected.
4. I understand that sections of any of my medical notes may be looked at by responsible investigators or delegates from regulatory authorities where it is relevant to my taking part in research. I give permission for these individuals to have access to my records.
5. I agree to take part in the above study.
6. I understand that I will receive a copy of the signed and dated consent form.

__________________________ _______________ _________________

Name of patient Date Signature

__________________________ _______________ _________________

Name of legally acceptable Date Signature

representative (if applicable)

__________________________ _______________ _________________

Name of witness (if applicable) Date Signature

__________________________ _______________ _________________

Researcher Date Signature

Copies to:

Patient/Subject

Researcher’s File
