# Supplementary material for: Identification of Chinese medicine syndromes in persistent insomnia associated with major depressive disorder: a latent tree analysis
Source: Chin Med. 2016 Feb 12;11:4. doi: 10.1186/s13020-016-0076-y (PMC4751631; doi:10.1186/s13020-016-0076-y)
Supplement: Supplementary file 4 — 10.1186/s13020-016-0076-y Chinese Medicine Insomnia Symptom Checklist. [file 13020_2016_76_MOESM4_ESM.doc]

**中醫失眠症狀表**

**Chinese Medicine Insomnia Symptom Checklist**

在過去兩星期, 你有沒有下列症狀呢 (如果病人有該症狀，則在方格裡填上√) ?

In the past 2 weeks, do you have the following symptoms (put a √ on the box if the patient has the symptom)?

請列出病人最先講出的三個主訴症狀

List the first 3 symptoms that the patient complains of:

1. ________________________________
2. ________________________________
3. ________________________________

| **睡眠症狀Sleep-related symptoms** | | | | **非睡眠症狀Non-sleep-related symptoms** | | |
| --- | --- | --- | --- | --- | --- | --- |
|  | Difficulty falling asleep | | 不易入睡 | **精神Vitality** | |  |
|  | Difficulty falling asleep alone | | 不能獨自安臥 |  | Dyspnea | 氣短 |
|  | Difficulty falling asleep with vexation | | 心煩難以入睡 |  | Fatigue | 乏力 |
|  | Difficulty staying asleep | | 易醒 |  | Lassitude | 神疲 |
|  | Excessive dreaming | | 多夢 |  |  |  |
|  | Frequent awakening with a start | | 易驚醒 | **望診Inspection** | |  |
|  | Half asleep | | 朦朧不實 |  | Reddish eyes | 目赤 |
|  | Insomnia | | 失眠 |  | Reddened cheeks | 顴紅 |
|  | Insomnia with vexation | | 心煩不寐 |  | Reddened complexion | 面紅 |
|  | Restless sleep | | 睡眠不安 |  | Reduction in luster complexion or lusterless complexion | 面色少華 |
|  | Shallow sleep | | 睡而不實 |  |  |  |
|  | Sleeping late at night | | 夜寐 | **頭面Head** | |  |
|  | Unrefreshing sleep | | 醒後困倦 |  | Headache | 頭痛 |
|  |  | |  |  | Head distension | 頭脹 |
| **非睡眠症狀Non-sleep-related symptoms** | | | |  | Heavy headedness | 頭重 |
| **飲食 Appetite** | | |  |  | Dizziness | 頭暈目眩 |
|  | Aphthous stomatitis | | 口舌生瘡 |  | Dizziness with headache | 頭暈脹痛 |
|  | Dry mouth | | 口乾 |  | Tinnitus | 耳鳴 |
|  | Bitter taste | | 口苦 |  |  |  |
|  | Dry throat | | 咽燥 | **四肢Limbs** | |  |
|  | Thirst | | 口渴 |  | Weary limbs | 腿困 |
|  | Favour of drinking | | 喜飲 |  | Sore knees | 腿軟 |
|  | Poor appetite | | 納差 |  | Backache | 腰酸 |
|  | Tasteless | | 飲食無味 |  |  |  |
|  | Profuse sputum | | 痰多 | **寒、熱、汗Cold, heat, and sweating** | |  |
|  | Belching | | 噯氣 |  | Cold extremities | 四肢發涼 |
|  | Acid regurgitation | | 吞酸 |  | Feverish sensations in the palms, soles and chest | 五心煩熱 |
|  | Nausea | | 噁心 |  | Hot flashes | 潮熱 |
|  |  | |  |  | Night sweating | 盜汗 |
|  |  | |  |  | Sweating | 出汗 |
| **二便 Urination and defecation** | | |  |  |  |  |
|  | Constipation | | 便秘 | **月經/男科Menstruation / andrology** | |  |
|  | Yellow urine | | 尿黃 |  | Menstrual disturbance | 月經不調 |
|  | Oliguria | | 小便短小 |  | Nocturnal emission | 夢遺 |
|  | Pale and large amount of urine | | 小便清長 |  | Seminal emission | 遺精 |
|  | Reddish urine | | 小便赤 |  | Spermatorrhea | 滑精 |
|  | Sloppy stool | | 便溏 | **情志Emotion** | |  |
| **胸腹Chest and abdomen** | | |  |  | Flusteredness | 心慌 |
|  | Stuffiness and pain in stomach and abdomen | | 脘腹脹痛 |  | Frequent sighing | 善太息 |
|  | Hypochondriac distension | | 兩脇脹痛 |  | Fright palpitation | 驚悸 |
|  | Hypochondriac pain | | 脇痛 |  | Thoughtful | 多慮 |
|  | Oppression in the chest | | 胸悶 |  | Impatience | 性情急躁 |
|  | Pain in the chest and hypochondrium | | 胸脇痛 |  | Irritability | 易怒 |
|  | Abdominal distention | | 腹脹 |  | Palpitation | 心悸 |
|  | Gastric stuffiness | | 脘痞 |  | Vexation | 心煩 |
|  | Stuffiness in stomach and abdomen | | 脘腹脹滿 |  | Poor memory | 健忘 |
|  |  | |  |  | Vexation in sitting and lying down | 坐臥不安 |
|  |  | |  |  | Susceptibility to fright | 遇事善驚 |
| **舌象 Tongue features** | | | | **脈象Pulse features** | | |
|  | Dry tongue | 舌燥 | |  | Fine pulse | 脈細 |
|  | No coating | 無苔 | |  | Rapid pulse | 脈數 |
|  | Pale tongue | 舌淡 | |  | Slippery pulse | 脈滑 |
|  | Red in the tip of tongue | 舌尖紅 | |  | String-like pulse | 脈弦 |
|  | Red tongue | 舌紅 | |  | Sunken pulse | 脈沉 |
|  | Scanty coating | 少苔 | |  | Strong pulse | 脈有力 |
|  | Slimy coating | 苔膩 | |  | Weak pulse | 脈象無力 |
|  | Thick coating | 苔厚 | |  |  |  |
|  | Thin coating | 苔薄 | |  |  |  |
|  | White coating | 苔白 | |  |  |  |
|  | Yellow coating | 苔黃 | |  |  |  |

其他症狀 Other symptoms: ______________________________________________________________________

**中醫證型診斷 (只可選以下其中一個)**

**TCM diagnosis (Check only 1 of the followings)**:

| 1. 心脾兩虛 □  *Deficiency of both the heart and spleen* | 2. 心腎不交 □*Heart-kidney noninteraction* | 3. 心膽氣虛 □  *Qi deficiency of the heart and gallbladder* | 4. 肝鬱化火 □*Liver-qi stagnation transforming into fire* | 5. 陰虛火旺 □*Hyperactivity of fire due to yin deficiency* | |
| --- | --- | --- | --- | --- | --- |
| 6. 痰熱內擾 □*Internal disturbance of phlegm-heat* | 7. 肝火上擾 □*Liver fire flaming upward* | 8. 胃腑不和 □*Stomach disharmony* | 9. 心虛膽怯 □  *Heart deficiency with timidity* | 10. 胃氣失和 □*Stomach qi disharmony* | |
| Others: _____________________________________________________________________________ | | | | |  |
