# Supplementary material for: Identification of Chinese medicine syndromes in persistent insomnia associated with major depressive disorder: a latent tree analysis
Source: Chin Med. 2016 Feb 12;11:4. doi: 10.1186/s13020-016-0076-y (PMC4751631; doi:10.1186/s13020-016-0076-y)
Supplement: Supplementary file 5 — 10.1186/s13020-016-0076-y Numbers (percentages) of manifestations of the subjects (N = 142). [file 13020_2016_76_MOESM5_ESM.docx]

**Supplementary Table 1**. Numbers (percentages) of manifestations of the subjects (N=142).

|  |  |  |  |  |  |
| --- | --- | --- | --- | --- | --- |
| Sleep related symptoms | N (%) | Tongue features | N (%) | Pulse features | N (%) |
| Difficulty falling asleep | 112 ( 78.9) | Scanty coating | 8 ( 5.6) | Fine pulse | 88 ( 62.0) |
| Difficulty falling asleep alone | 9 ( 6.3) | Slimy coating | 16 ( 11.3) | Rapid pulse | 16 ( 11.3) |
| Difficulty falling asleep with vexation | 63 ( 44.4) | Thick coating | 13 ( 9.2) | Slippery pulse | 95 ( 66.9) |
| Difficulty staying asleep | 121 ( 85.2) | Thin coating | 77 ( 54.2) | String-like pulse | 76 ( 53.5) |
| Excessive dreaming | 93 ( 65.5) | White coating | 71 ( 50) | Sunken pulse | 42 ( 29.6) |
| Frequent awakening with a start | 35 ( 24.6) | Yellow coating | 37 ( 26.1) | Strong pulse | 2 ( 1.4) |
| Half asleep | 65 ( 45.8) | Dry tongue | 10 ( 7.0) | Weak pulse | 3 ( 2.1) |
| Insomnia | 95 ( 66.9) | No coating | 9 ( 6.3) |  |  |
| Insomnia with vexation | 33 ( 23.2) | Pale tongue | 77 ( 54.2) |  |  |
| Restless sleep | 88 ( 62.0) | Red in the tip of tongue | 18 ( 12.7) |  |  |
| Shallow sleep | 110 ( 77.5) | Red tongue | 47 ( 33.1) |  |  |
| Sleeping late at night | 59 ( 41.5) |  |  |  |  |
| Unrefreshing sleep | 123 ( 86.6) |  |  |  |  |

**Supplementary Table 1 (Cont’)**. Numbers (percentages) of manifestations of the subjects (N=142).

| Other clinical features | N (%) | Other clinical features | N (%) | Other clinical features | N (%) |
| --- | --- | --- | --- | --- | --- |
| Aphthous stomatitis | 46 ( 32.4) | Reduction in luster complexion or lusterless complexion | 34 ( 23.9) | Hypochondriac pain | 17 ( 12.0) |
| Dry mouth | 112 ( 78.9) | Headache | 78 ( 54.9) | Oppression in the chest | 45 ( 31.7) |
| Bitter taste | 69 ( 48.6) | Head distension | 53 ( 37.3) | Reddish eyes | 3 ( 2.1) |
| Dry throat | 50 ( 35.2) | Heavy headedness | 44 ( 31.0) | Reddened cheeks | 5 ( 3.5) |
| Thirst | 85 ( 59.9) | Dizziness | 41 ( 28.9) | Reddened complexion | 6 ( 4.2) |
| Favour of drinking | 74 ( 52.1) | Dizziness with headache | 21 ( 14.8) | Pain in the chest and hypochondrium | 8 ( 5.6) |
| Poor appetite | 34 ( 23.9) | Tinnitus | 52 ( 36.6) | Abdominal distention | 25 ( 17.6) |
| Tasteless | 16 ( 11.3) | Weary limbs | 81 ( 57.0) | Gastric stuffiness | 29 ( 24) |
| Profuse sputum | 37 ( 26.1) | Sore knees | 63 ( 44.4) | Stuffiness in stomach and abdomen | 41 ( 28.9) |
| Belching | 80 ( 56.3) | Backache | 92 ( 64.8) | Flusteredness | 78 ( 54.9) |
| Acid regurgitation | 51 ( 35.9) | Cold extremities | 53 ( 37.3) | Frequent sighing | 62 ( 43.7) |
| Nausea | 18 ( 12.7) | Feverish sensations in the palms, soles and chest | 33 ( 23.2) | Fright palpitation | 25 ( 17.6) |
| Constipation | 42 ( 29.6) | Hot flashes | 45 ( 31.7) | Thoughtful | 106 ( 74.6) |
| Yellow urine | 48 ( 33.8) | Night sweating | 45 ( 31.7) | Impatience | 85 ( 59.9) |
| Oliguria | 16 ( 11.3) | Sweating | 56 ( 39.4) | Irritability | 62 ( 43.7) |
| Pale and large amount of urine | 22 ( 15.5) | Menstrual disturbance | 29 ( 24) | Palpitation | 30 ( 21.1) |
| Reddish urine | 3 ( 2.1) | Nocturnal emission | 0 ( 0) | Vexation | 58 ( 48) |
| Sloppy stool | 31 ( 21.8) | Seminal emission | 0 ( 0) | Poor memory | 126 ( 88.7) |
| Dyspnea | 45 ( 31.7) | Spermatorrhea | 0 ( 0) | Vexation in sitting and lying down | 9 ( 6.3) |
| Fatigue | 80 ( 56.3) | Stuffiness and pain in stomach and abdomen | 29 ( 24) | Susceptibility to fright | 28 ( 19.7) |
| Lassitude | 123 ( 86.6) | Hypochondriac distension | 12 ( 8.5) |  |  |

**Supplementary Table 2**. Percentages of each cluster in the latent variables.

| Latent variables | Percentage of subjects classified as the cluster Y = s0 | Percentage of subjects classified as the cluster Y = s1 |
| --- | --- | --- |
| Y01 | 48% | 52% |
| Y02 | 54% | 46% |
| Y03 | 79% | 21% |
| Y04 | 76% | 24% |
| Y05 | 55% | 45% |
| Y06 | 31% | 69% |
| Y07 | 39% | 61% |
| Y08 | 39% | 61% |
| Y09 | 46% | 54% |
| Y10 | 61% | 39% |
| Y11 | 88% | 12% |
| Y12 | 48% | 52% |
| Y13 | 82% | 18% |
| Y14 | 69% | 31% |
| Y15 | 81% | 19% |
| Y16 | 62% | 38% |
| Y17 | 34% | 66% |
| Y18 | 43% | 57% |
