# Supplementary material for: Identification of Chinese medicine syndromes in persistent insomnia associated with major depressive disorder: a latent tree analysis
Source: Chin Med. 2016 Feb 12;11:4. doi: 10.1186/s13020-016-0076-y (PMC4751631; doi:10.1186/s13020-016-0076-y)
Supplement: Supplementary file 1 — 10.1186/s13020-016-0076-y Research ethic approval. [file 13020_2016_76_MOESM1_ESM.pdf]

香港大學
University of Hong Kong

醫院管理局
HOSPITAL AUTHORITY

香港大學及醫管局港島西醫院聯網研究倫理委員會

Institutional Review Board of the University of Hong Kong/ Hospital Authority Hong Kong West Cluster (HKU/HA HKW IRB)

Address: Rm 901, Administration Block, QMH Tel 2855 3923 2855 4086 Fax 2855 4735

Terry, please [illegible] J

Dr. KF Chung
Psychiatry
Queen Mary Hospital
31-Dec-08

Dear Dr. Chung,

IRB Reference Number: **UW 08-417**

The HKU/HA HKW IRB is authorized by a joint agreement of the University of Hong Kong and Hospital Authority Hong Kong West Cluster to review and monitor clinical research. It serves to ensure that research complies with the Declaration of Helsinki and acts in accordance to ICH GCP guidelines, local regulations and Hospital Authority and the University policies.

I write to inform that your research application has been approved by a full review with details shown below. You are also requested to adhere to the conditions listed.

Protocol title : A randomized controlled trial of electroacupuncture for residual insomnia associated with major depressive disorder

Study site(s) : As stated in application form

Date of full review : 23-12-2008 (Date/Month/Year)(Membership of the review panel is listed at the end of this letter in note 1)

Documents approved :
: 01. Clinical research ethics review application form
: 02. Research protocol
: 03. Patient information sheet and consent form (VER 0.9 21/9/2008) - English and Chinese, revised on 19.12.2008
: 04. Sleep log - Chinese
: 05. Hamilton depression rating scale (HDRS) - English
: 06. Insomnia severity index (ISI) - Chinese
: 07. Pittsburgh sleep quality index (PSQI) - Chinese
: 08. Sheehan disability index (SDI) - Chinese
: 09. Credibility of treatment rating scale - Chinese
: 10. Advertisement - Chinese

Documents reviewed : 11. Short CV of principal investigator

(Conditions :
1. Apply a clinical trial certificate from Department of Health if indicated.
2. Do not deviate from, or make changes to the study protocol without prior written IRB approval, except when it is necessary to eliminate immediate hazards to research subjects or when the change involves only logistical or administrative issues.
3. Report the following to HKU/HA HKW IRB: (i) study protocol or consent document change (use 'HKU/HA HKW IRB RE001F7'), (ii) serious adverse event (use 'HKU/HA HKW IRB RE001F8'), (iii) study progress (use 'HKU/HA HKW IRB RE001F9a'), and (iv) new information that may be relevant to a subject's willingness to continue participation in the study.
4. Report first study progress to HKU/HA HKW IRB at a 12-monthly interval until study closure.

5. For Phase I, II, III clinical trials, the study should only commence after the Sponsor has provided an indemnity satisfying the Hospital Authority's requirement.)

Yours sincerely,

W. H. Lee
Secretary, HKU/HA HKW IRB

(Note 1: IRB Review Panel of the meeting held on : 23-Dec-08

Membership		Name	Position/Affiliation	Gender
Chairman	Prof.	Poon, Ronnie	Professor, Surgery, HKU	M
Member	Dr.	Chung, Hon Ping	Consultant, Surgery, TWH	M
	Dr.	Jim, Man Hong	Associate Consultant, Cardiac Medicine, GH	M
	Prof.	Lam, Cindy	Professor, Family Medicine, HKU	F
	Dr.	Lee, Jospeh	Hospital Chief Executive, GH	M
	Dr.	Tang, Sydney	Associate Professor, Medicine, HKU	M
Lay member	Mr.	Wong, Paul	Retired Person - No affiliation with HKU and HKW Cluster Hospitals	M
